# Myocardial injury before noncardiac surgery

**DOI:** 10.3389/fcvm.2023.1207124

**Published:** 2023-08-25

**Authors:** Zhibing Chen, Yitao Zhang, Weijie Zeng, Lin Ye, Changda Yu, Fan Shi

**Affiliations:** ^1^ Department of General Surgery, Jiujiang First People’s Hospital, JiuJiang, China; ^2^ Cardiovascular Department, The Sixth Affiliated Hospital of Sun Yat-sen University, Guangzhou, China; ^3^ Internal Medicine-Cardiovascular Department, Jiujiang First People’s Hospital, JiuJiang, China

**Keywords:** perioperative period, noncardiac surgery, troponin, acute coronary syndrome, decision making

## Abstract

Non-cardiac surgical procedures present a significant circulatory stress and can potentially trigger cardiovascular events, such as myocardial infarction and heart failure. Myocardial injury before non-cardiac surgery is associated with an increased risk of mortality and major cardiovascular complications during perioperative period, as well as up to 5 years after non-cardiac surgery. While the definition of preoperative myocardial injury is not yet clear, it is generally understood as myocardial injury resulting from various causes of troponin elevation without acute coronary syndrome prior to surgery. Detecting preoperative myocardial injury through routine troponin monitoring is crucial for reducing perioperative risk, but it is also challenging. The aim of this review is to discuss the definition of preoperative myocardial injury, its pathophysiology, implications on clinical practice and decision-making for patients with elevated troponin levels before non-cardiac surgery.

## Introduction

Major surgical procedures are performed on over 300 million patients worldwide annually, accounting for about 5% of the global population. Between 2004 and 2012, there was a 34% increase in the number of such procedures, with nearly 85% of them were non-cardiac surgical procedures. As the prevalence of coronary artery disease (CAD), valvular heart disease (VHD), heart failure, and arrhythmias rises with age, perioperative cardiovascular mortality and morbidity primarily affect adults undergoing major non-cardiac surgical procedure (1).

Non-cardiac surgical procedures present a significant circulatory stress and can potentially trigger cardiovascular events, including myocardial infarction and heart failure (2). To prevent and minimize such events, it is crucial to predict the occurrence of major adverse cardiovascular events (MACE) before surgery. While several scales and scores are available to assess perioperative cardiovascular risk, they may no longer meet clinical needs. Recent studies have shown that elevated preoperative troponin levels are strongly linked to perioperative cardiovascular risk and have a particularly robust predictive value for postoperative mortality in patients undergoing non-cardiac surgery (3–8). However, implementing routine preoperative troponin monitoring worldwide presents several challenges. Firstly, many patients with elevated high-sensitivity troponin do not meet the diagnostic criteria for myocardial infarction (9–11). Secondly, the predictive value of preoperative high-sensitivity troponin on perioperative MACE has not received much attention from most surgeons. Lastly, there is no clear management strategy for patients with elevated high-sensitivity troponin or preoperative myocardial injury. Consequently, further understanding of the underlying causes of myocardial injury before non-cardiac surgery is essential to enhance patient management and outcomes. This article aims to explore the definition, etiology, impact, and potential prevention and management strategies for myocardial injury prior to non-cardiac surgery.

### Scope of the problem

Different from the definition of myocardial injury after non-cardiac surgery (MINS) (12), there is no clear definition of myocardial injury before noncardiac surgery. However, most clinical studies on preoperative myocardial injury included myocardial ischemia caused by non-coronary lesions and excluded type 1 myocardial infarction (6, 10, 11).

Over the past few years, several studies have assessed specific high-sensitivity troponin assays for their potential to enhance the triage of patients suspected of having acute myocardial infarction (AMI) (13). Earlier studies have confirmed the predictive value of high-sensitivity troponin for adverse cardiovascular events in patients with stable coronary artery disease, chronic heart failure, and even in the general population (14–18). According to the 2022 ESC/ESA guidelines, routine monitoring of high-sensitivity troponin is recommended for patients with known cardiovascular disease (CVD), cardiovascular risk factors (including age ≥65 years), or symptoms suggestive of CVD (1).

The guidelines recommend the use of both troponin T and troponin I for routine monitoring, which is common practice in clinical settings. A prospective study comparing preoperative high-sensitivity cardiac troponin I (hs-cTnI) and T (hs-cTnT) for the prediction of cardiac complications after non-cardiac surgery showed that hs-cTnI and hs-cTnT concentrations predict major cardiac complications after non-vascular surgery, while hs-cTnI may be more accurate in patients undergoing vascular surgery (19). Another study involving 19,501 patients with a mean follow-up of 7.8 years found that elevations in cTnI are more strongly associated with certain cardiovascular disease outcomes, whereas cTnT is more strongly linked to non-cardiovascular death risk in the general population (20). As troponin is used as a screening tool in postoperative monitoring for patients without symptoms, there is currently no evidence indicating that one assay (T or I) is preferable over the other.

The 2022 ESC/ESA guidelines do not define a threshold to be used in the preoperative setting, and previous studies did not establish a definitive cut-off value either. As a result, hospitals should rely on the clinical threshold applied in their respective clinics, typically defined as a value exceeding the 99th percentile of a normal reference population as recommended in the fourth universal definition of myocardial infarction (9).

### Pathophysiology

In clinical practice, the causes of elevated troponin levels are typically categorized into 3 groups: myocardial damage related to supply-demand mismatch, myocardial damage related to non-ischemic causes and multifactorial or indeterminate cause myocardial injury (21). For example, persistently elevated troponin levels are prevalent in chronic kidney disease (CKD) patients in the absence of clinical evidence of acute myocardial infarction (AMI) (14, 22). A meta-analysis found increased serum troponin levels predict worse long-term cardiovascular outcomes and poor survival in asymptomatic patients with CKD in the absence of AMI, higher values are associated with a worse prognosis (23). Classic chemotherapy drugs such as anthracyclines and fluorouracil can also cause cardiomyocyte damage (24). A single-center study with 2,285 subjects receiving anthracycline-based chemotherapy found that patients with a baseline left ventricular ejection fraction (LVEF) ≤5 points above the lower limit of normal have higher incidence of MACE (25). Another retrospective study found gastrointestinal tumor patients with elevated hs-cTnI without evidence of myocardial ischemia meet more in-hospital all-cause mortality, acute myocardial infarction, cardiac arrest or ventricular fibrillation and acute decompensated heart failure (11). Type A aortic dissection, trauma, especially severe trauma (cardiac contusion), as well as surgery or catheter-based cardiac interventions, can all potentially cause elevated levels of cardiac troponin, and their complications may lead to non-cardiac surgery (26, 27). Table 1 shows the most common cause of an elevated troponin apart from type 1 myocardial infarction (9).

**Table 1:** Causes of elevated troponin prior to non-cardiac surgery not associated with type 1 myocardial infarction.

Cardiac pathologies
Coronary ischemia
Cardiac arrhythmias (tachycardia or bradycardia)
Coronary artery spasm
Stable coronary atherosclerotic disease in setting of increased o_2_ demand
Severe hypertension
Coronary artery vasculitis
Coronary embolus
Aortic dissection
Myocardial damage
Cardiotoxic meds (eg, anthracyclines, herceptin et al)
Electrical shock
Cardiac contusion
Cardiomyopathy
Takotsubo syndrome
Myocarditis
Myopericarditis
Rhabdomyolysis involving cardiac muscle
Cardiomyopathy
Heart failure
Malignancy
Cardiothoracic surgery (valve replacement, valve repair, catheter ablation, etc.)
Non-cardiac pathologies
Comorbidities
Renal failure
Acute respiratory failure
Subarachnoid hemorrhage
Stroke
Infiltrative diseases
Sepsis
COVID-19
Specific identifiable precipitants
Extreme exertion
Burns >30% body surface area
Heavy physical activity
Noncoronary ischemia
Shock
Hypoxia
Hypoperfusion
Pulmonary embolism
Global ischemia

The mechanism of myocardial injury can be mainly attributed to the following 4 aspects: (1) myocytes apoptosis, programmed cell death, which leads to rapid uptake by scavenging macrophages before significant cellular contents are released. In healthy individuals with normal cellular turnover and apoptosis, only low levels of troponin would be present in the serum. While older assays may not detect these low levels, newer high-sensitivity assays can likely detect them (28). (2) Infarction or ischemic myocyte necrosis, myocytes undergo irreversible damage (necrosis) with prolonged ischemia, resulting in the degradation of the cell membrane and the gradual release of myofibril-bound cytosolic complexes. Additionally, brief periods of ischemia, sudden increases in preload, and physiological challenges such as tachycardia and catecholamines can also lead to the release of cTn (29, 30). (3) Increased myocyte membrane permeability, it is believed that myocardial depressive factors released in the context of sepsis and other inflammatory states may cause the degradation of free troponin to lower-molecular-weight fragments. With increased membrane permeability, smaller troponin fragments may be released into the systemic circulation. In this scenario, troponin levels may be elevated, despite the absence of myocyte cell death (31). (4) Nonischemic myocyte necrosis, this type of necrosis is triggered by oxidative stress, reactive oxygen species, inflammatory cytokines, neurohormonal activation, altered calcium handling, and acid-base disturbances. Increased preload, which alters calcium handling, activates intracellular proteolytic enzymes that degrade cardiac troponin (cTn), releasing cTn fragments into the bloodstream. These fragments may contain epitopes that bind to cTnI immunoassays (28).

### Implications on clinical practice

Certain patients undergoing non-cardiac surgery face a heightened risk of experiencing negative cardiovascular events. The risk extent is influenced by individual patient factors and the nature of the surgery. Identifying those at higher risk can assist the patient, anesthesiologist, and surgeon in comprehending the potential benefits and risks of a procedure, and may lead to implementing interventions that reduce the chances of adverse events. Several tools or indices are available for predicting perioperative risk. Guidelines recommend using a combination of the Revised Cardiac Risk Index (RCRI), an online calculator based on the National Surgical Quality Improvement (NSQIP) database, or the American College of Surgeons Surgical Risk Calculator (1). Regrettably, none of the scoring systems mentioned above include brain natriuretic peptide (BNP) or troponin in their calculations.

For planned non-cardiac surgery, Gibson et al. were the first to discover that an increase in pre-operative cardiac troponin I (cTnI) levels was strongly linked to adverse outcomes and was the only significant predictor of post-operative cardiac events in patients undergoing major lower extremity amputation (32). Since then, some clinical studies on vascular surgery have confirmed this conclusion (3, 5). Moreover, studies involving other types of non-cardiac surgical procedures have also established the predictive value of preoperative troponin levels for perioperative outcomes including perioperative MACE, hospital stay, chance of intensive care, and mortality (4, 8, 33, 34). In addition, a prospective, international multicenter observational study that enrolled nearly 1,000 patients also demonstrated that preoperative high-sensitive troponin T provides strong prognostic information in patients undergoing non-cardiac surgery incremental to the widely accepted revised cardiac index (6, 35). Another large retrospective study conducted at a single center, focusing on gastrointestinal tumor surgical procedures, also supported this conclusion (11) and found elevated hs-cTnI prior to tumor resection surgery were at increased risk for long-term all-cause death and MACE (10).

In contrast to patients undergoing elective surgery, those with acute surgical diagnoses frequently encounter substantial physiological stress before their surgical intervention. Zimmerman, A. M and colleagues first found emergency general surgery patients who experience preoperative myocardial injury face a heightened risk of postoperative events and mortality. Preoperative myocardial injury serves as an independent predictor of death (8). Ma, Jinling and colleagues also confirmed preoperative plasma cTnI was independently associated with an increased risk of MACE in elderly patients undergoing emergency surgery (36).

Table 2 summarizes recent research on the relationship between preoperative troponin levels and prognosis in non-cardiac surgical procedures (38, 39–41). These findings emphasize the significance of incorporating preoperative troponin levels into the perioperative risk assessment system for non-cardiac surgical procedures, regardless of whether they are emergency or non-emergency procedures.

**Table 2:** Studies on the relationship between preoperative troponin levels and prognosis.

Study	Year	Sample size	Study type	Surgery type	Follow-up time
Gibson et al. (32)	2006	44	Prospective blinded observational study	Lower extremity amputation	6 weeks
Biccard et al. (3)	2012	560	Prospective observational study	Elective vascular surgery	30 days
Thielmann et al. (37)	2012	46	Retrospective single-centre study	Acute surgical pulmonary embolectomy	30 days
Nagele et al. (33)	2013	599	Prospective cohort study	Major non-cardiac surgery	3 years
Weber et al. (6)	2013	979	Prospective international multi-centre observational study	Major non-cardiac surgery	Duration of hospitalization
Gillmann et al. (5)	2014	455	Prospective non-interventional trial	Open vascular surgery	30 days
Ma et al. (36)	2015	2,519	Prospective observational study	Emergent non-cardiac surgery	30 days
Maile et al. (4)	2016	6,030	Single-institution retrospective cohort study	Non-cardiac surgery	30 days
Zimmerman et al. (8)	2016	464	Retrospective review using NSQIP data	Emergency general surgery	30 days
Gualandro et al. (19)	2018	1,022	Prospective cohort study	Non-cardiac surgery	30 days
Golubović et al. (38)	2018	79	Prospective single-center observational study	Major noncardiac surgery	14 days
Hao et al. (39)	2020	789	Prospective cohort study	Total knee arthroplasty	2 years
Zhang et al. (11)	2021	1,259	Single institution, retrospective cohort study	Gastrointestinal tumor surgery	Duration of hospitalization
Zhang et al. (10)	2023	1,105	Single institution retrospective cohort study	Colorectal tumor resection surgery	24.4 ± 10.8 months
Park et al. (40)	2023	703	Single-institution, retrospective cohort study	Non-cardiac surgery	30 days
Zhu et al. (41)	2023	7,156	Single-institution, retrospective cohort study	Non-cardiac surgery	1 years

Perioperative MACE including myocardial infarction, arrhythmia and heart failure, has been described in detail by Sellers and colleagues (2), and it is noteworthy why these events increased in patients with preoperative troponin elevations. The main reasons may be: The latest high-sensitivity assays allow for the accurate detection of low concentrations of troponin, even in apparently healthy individuals. Moreover, patients with cardiovascular disease other than acute coronary syndrome may have slightly elevated troponin levels. Additionally, low levels of troponin elevation have been detected in stable patients and non-acute subjects, indicating mild myocardial injury (6). During the perioperative period, environmental changes may exacerbate this mild myocardial injury, leading to conditions such as myocardial infarction, heart failure, or arrhythmia.

Long-term MACE is a combined endpoint, including cardiac death, myocardial infarction, heart failure, atrial fibrillation, cardiac arrest, cardiogenic shock and other related events. Due to limited sample sizes, most relevant studies have used MACE (major adverse cardiovascular events) as the primary research endpoint rather than individual cardiovascular disease events such as heart failure and atrial fibrillation. Thus, it remains uncertain whether there are statistically significant differences in these individual cardiovascular events. Furthermore, the association between troponin levels and the risk of long-term cardiovascular events in individuals without pre-existing cardiovascular risk factors prior to non-cardiac surgery remains unclear, and additional research is necessary to investigate this group in the future.

### Implications on decision making

**Figure 1:**
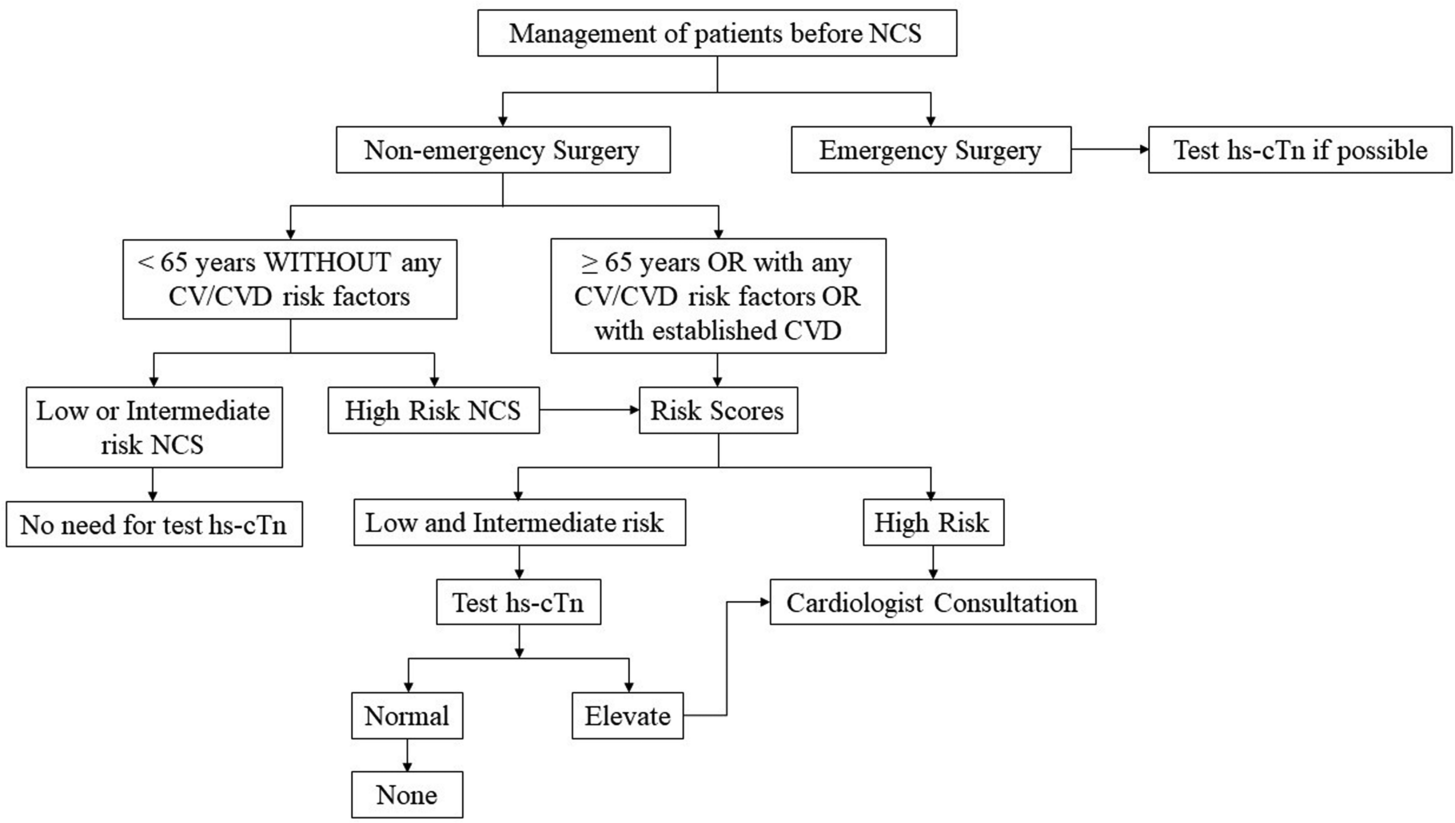
Flow chart about decision making process in patients with and without elevated troponins. NCS, non-cardiac surgery; hs-cTn, high-sensitivity cardiac troponin; CV, cardiovascular; CVD, cardiovascular disease.

Elevated preoperative troponin levels can increase the risk of cardiovascular complications during non-cardiac surgery. Given the predictive value of preoperative troponin levels on perioperative outcomes, it is justifiable to classify patients with elevated troponin levels as a high-risk group. Therefore, in high-risk patients, the predictive value of troponin detection for perioperative cardiovascular risk appears to be inferior compared to that in low- and medium-risk groups. For high-risk groups, it is advisable to consider more advanced imaging examinations instead of relying solely on troponin measurement for risk screening, as shown in Figure 1. Our previous research also supports this perspective (11). Consequently, clinical decision-making for these patients primarily revolves around reducing perioperative cardiovascular risk. The 2022 ESC Guidelines provide detailed strategies to mitigate perioperative risk (1), which we will not reiterate here. Patients with elevated preoperative troponin levels require further consideration in 4 areas: (1) Whether there is a need for coronary assessment and, if so, what type of assessment is appropriate; and (2) Whether a cardiologist is needed to assist in the assessment; (3) Whether pharmacological intervention is necessary and which medications to use for intervention; (4) Exploring additional methods beyond existing guidelines to reduce perioperative risk.

The evaluation of myocardial ischemia involves various imaging tests, such as exercise stress testing, stress imaging, coronary computed tomography angiography, and invasive coronary angiography. Of these, exercise stress testing is of limited diagnostic value in patients with pre-existing ST-segment abnormalities and is unsuitable for individuals with limited exercise capacity. Additionally, its sensitivity (61%–73%) and specificity (60%–80%) are suboptimal. As a result, exercise stress testing is generally not the preferred recommendation for assessing myocardial ischemia (1). According to the 2022 ESC guidelines, stress imaging is recommended before high-risk elective non-cardiac surgery for patients with poor functional capacity and a high likelihood of coronary artery disease (CAD) or high clinical risk. However, it should be noted that stress imaging exhibits lower sensitivity and specificity when compared to coronary computed tomographic angiography (CCTA). Due to the absence of data from randomized controlled trials (RCTs) concerning the utility of invasive coronary angiography (ICA) in patients scheduled for non-cardiac surgery (NCS), the 2022 ESC guidelines do not provide specific recommendations regarding the selection between ICA and CCTA. However, adopting an ICA assessment may also cause an unnecessary and unpredictable delay in an already planned surgical intervention, and adding an independent procedural risk to the overall risk. Several prospective multicenter studies have provided evidence regarding the diagnostic accuracy of CCTA in patients with suspected but unconfirmed CAD. These studies have reported a sensitivity ranging between 85% and 99% and a specificity between 64% and 92% for CCTA in this patient population (42–44). Moreover, significant advancements in relevant technologies have led to improved diagnostic accuracy in detecting significant coronary artery stenosis (defined as ≥50% luminal narrowing). These advancements have proven effective even in patients with conditions such as atrial fibrillation and/or a high heart rate (45). As a result, CCTA is suggested as the initial test for diagnosing CAD in stable patients with a low clinical likelihood or no previous diagnosis of CAD (46, 47).

Anesthesiologist and surgeons may often seek the assistance of a cardiologist to evaluate cardiovascular risk. However, some studies have showed that preoperative cardiology consultation not only provides little advice that impacts perioperative outcome but also increased cardiac testing, the length of stay, and financial burden (48, 49). Therefore, the prudent utilization of preoperative consultation and investigations is essential to avoid unnecessary delays in surgery, reduce healthcare costs, and promote cost-effective healthcare delivery.

Furthermore, the choice of anesthesia is another aspect that requires thorough exploration. A multicenter, single-blind, controlled trial conducted in 36 centers across 13 countries revealed that among patients undergoing elective coronary artery bypass grafting (CABG), anesthesia with a volatile agent did not result in significantly fewer deaths at 1 year than total intravenous anesthesia (50). Nevertheless, the controversy persists regarding whether volatile anesthetics provides better outcomes for patients with preoperative myocardial injury during noncardiac surgery compared to total intravenous anesthesia. In a single center, retrospective cohort study of 1,254 patients with preoperative myocardial injury undergoing non-cardiac surgery, the researchers found the use of volatile anesthetics showed the significant survival improvement after non-cardiac surgery in patients with preoperative myocardial injury (51). However, a different study focusing on abdominal great vessel surgery did not yield positive results (52). Hence, further large multicenter, prospective studies are needed to establish whether volatile anesthetics offer beneficial effects.

## Conclusions

Elevated preoperative troponin levels predict adverse cardiovascular events in non-cardiac surgery. Understanding the multifactorial mechanisms behind myocardial injury is crucial. Embracing troponin monitoring enhances perioperative outcomes and patient safety. Integrating troponin assessment in clinical practice aids risk identification and tailored management. Further research is needed for clear management strategies.
